# BMS-690514, a VEGFR and EGFR tyrosine kinase inhibitor, shows anti-tumoural activity on non-small-cell lung cancer xenografts and induces sequence-dependent synergistic effect with radiation

**DOI:** 10.1038/sj.bjc.6605748

**Published:** 2010-07-13

**Authors:** Y Loriot, P Mordant, N Dorvault, T De la motte Rouge, J Bourhis, J-C Soria, E Deutsch

**Affiliations:** 1 Institut Gustave Roussy, UPRES 27-10, 39 rue Camille Desmoulins, 94800 Villejuif, France; 2 University Paris-Sud, 63, rue Gabriel Péri, 94276 Le Kemlin-Bicêtre Cedex, France; 3 Department of medicine, Institut Gustave Roussy, 94800 Villejuif, France

**Keywords:** radiation, non-small cell lung cancer, VEGFR inhibitor, EGFR inhibitor

## Abstract

**Background:**

Non-small-cell lung cancer (NSCLC) is an aggressive disease in which vascular endothelial growth factor (VEGF) and epidermal growth factor (EGF) are implicated in tumour growth, tumour resistance to radiation and chemotherapy, and disease relapse. We have investigated the anti-tumoural effects of BMS-690514, an inhibitor of both vascular endothelial growth factor receptor (VEGFR) and epidermal growth factor receptor (EGFR) signalling pathways, as a single agent and in combination with ionising radiation (IR) on several NSCLC cell lines.

**Methods:**

Radiosensitisation of several NSCLC cell lines by BMS-690514 was assessed *in vitro* using clonogenic assay and *in vivo* using nude mice.

**Results:**

*In vitro* studies showed that BMS-690514 alone decreases clonogenic survival of NSCLC cells lines but no potential enhancement of IR response was observed in the combination. In tumour-bearing mice, BMS-690514 alone inhibits the growth of NSCLC xenografts, including the T790M mutation-harbouring H1975 tumour. The concomitant combination of BMS-690514 and radiation did not increase mice survival in comparison with treatment with IR alone. In contrast, BMS-690514 markedly enhances the anti-tumour effect of radiation in a sequential manner on H1299 and H1975 xenografts. Immunohistochemistry revealed a qualitative reduction in vessel area after administrations of BMS-690514, compared with vehicle-treated controls, suggesting that revascularisation may explain the schedule dependency of the tumour-growth delay observed.

**Conclusion:**

The results of association with radiation show that BMS-690514 may be a successful adjuvant to clinical radiotherapy. These findings are of translational importance because the clinical benefits of anti-EGFR and anti-VEGFR therapy might be schedule dependent.

Lung cancer is a major health problem worldwide and is the leading cause of cancer-related death for men and women (Alberg *et al*, 2005). Non-small-cell lung cancer (NSCLC) accounts for 85% of lung cancer and patients typically present with locally advanced or metastatic disease (Toschi *et al*, 2007). Currently, treatment options for a patient with locally advanced lung cancer include chemotherapy, radiotherapy, and/or surgery (Fossella *et al*, 2003). Unfortunately, recent studies demonstrate that conventional therapies lead to a poor outcome, with a 5-year survival rate for NSCLC that remains at 15% (Toschi *et al*, 2007). Hence, the current therapeutic challenge is to optimise non-operative strategies by incorporating new agents into current therapeutic regimens. Targeting the tumour vasculature with anti-angiogenic agents in combination with radiotherapy is one current strategy (O’Reilly, 2006).

Inhibiting the epidermal growth factor (EGF) pathway is another promising approach for improving the anti-tumoural activity of radiation. A pivotal study reported a benefit in overall survival and locoregional control in patients with localised head and neck cancer who were treated with both radiation and cetuximab, a monoclonal antibody against epidermal growth factor receptor (EGFR), over patients treated with radiation alone (Bonner *et al*, 2006). Several studies demonstrated that angiogenesis and the expression of vascular endothelial growth factor (VEGF) and other pro-angiogenic factors and their receptors correlate with a less favourable clinical outcome for lung cancer patients; yet clinical trials of agents that target these pathways alone have been disappointing (Bremnes *et al*, 2006). Preclinical studies suggest that monotherapy with anti-angiogenic agents will not be sufficient for the treatment of advanced cancer (O’Reilly *et al*, 1994; Boehm *et al*, 1997), given that tumours typically progress before they respond to therapy and microscopic residual disease persists even after prolonged therapy with these agents. In clinical trials, bevacizumab, a humanised antibody against VEGF, has shown considerable promise and improved survival in patients with colorectal, breast, or lung cancer when used in combination with chemotherapy (Hurwitz *et al*, 2004; Miller *et al*, 2005; Sandler *et al*, 2006).

Several findings argue in favour of combining radiation and an anti-angiogenic inhibitor (Senan and Smit, 2007).

Radiation induces transient tumour hypoxia, which in turn stimulates VEGF production and vascular endothelial growth factor receptor-2 (VEGFR-2) expression (Gorski *et al*, 1999). Vascular endothelial growth factor protects endothelial cells from radiation-induced cytotoxicity (Kermani *et al*, 2001), and vasculature damage decreases tumour cell survival (Garcia-Barros *et al*, 2003). After radiotherapy or chemoradiotherapy, a subset of patients fail to achieve long-term local tumour control. Hypoxia or hypoxia-inducible factor-1 expression is associated with a lower radiation response and malignant progression in tumours of the uterine cervix, head and neck, as well as in sarcomas, suggesting that angiogenesis could be involved in radioresistance (Hirota and Semenza, 2006).

Preclinical studies have demonstrated enhanced radiation-induced cell kill when anti-angiogenic therapy is combined with radiotherapy (Lee *et al*, 2000; Hess *et al*, 2001). The normalisation of tumour vasculature by an anti-VEGFR-2 antibody creates a time period of increased oxygenation, during which enhanced radiation-induced regression is observed (Winkler *et al*, 2004).

Combination of anti-angiogenic therapies and radiotherapy may be effective against tumour stem cells that have self-renewing potential, quiescence of cell cycling, and relative resistance to growth factors (Baumann *et al*, 2008).

We hypothesised that targeting both the tumour and its vasculature by VEGFR and EGFR blockade would improve lung cancer treatment, particularly when combined with radiation therapy. To test this hypothesis, we evaluated the feasibility of combining radiation with BMS-690514, a potent, orally administrable inhibitor of VEGFR and EGFR tyrosine kinase activity that was shown to have *in vitro* anti-tumour activity against lung cancer cells in a panel of human NSCLC *in vitro* experiments (De La Motte Rouge *et al*, 2007). The aim of this study was to evaluate the effects of EGFR and VEGFR signalling inhibition on the response to radiotherapy, to determine whether scheduling of BMS-690514 and ionising radiation (IR) affected anti-tumour efficacy, to assess the influence of EGFR mutation on tumour response, and to investigate whether the effects of the treatments were related to changes in tumour perfusion and vasculature. In this study, we report that BMS-690514 has *in vivo* efficacy on a panel of NSLCC xenografts, including those with T790M mutations, conferring resistance to EGFR therapies such as Erlotinib or Gefitinib. Furthermore, adjuvant administration of BMS-690514 was able to markedly enhance tumour response to IR *in vivo* in H1299 and H1975 xenografts.

## Materials and methods

### Cell lines, culture, and treatments

A549 cells (wild-type (WT) EGFR and p53) were grown in F12-K medium containing L-glutamine, supplemented with 10% foetal calf serum, 100 U ml^−1^ penicillin-G sodium, and 100 *μ*g ml^−1^ streptomycin sulphate. H1299 cells (WT EGFR, inactive p53 R175H), H1650 cells (bearing a deletion in exon 19 of the *EGFR* gene, that is, ΔE746–A750 and WT p53), and H1975 cells (EGFR L858R/T790M and WT p53) were maintained in RPMI 1640 with GlutaMAX supplemented with 10% foetal calf serum and antibiotics (as above). Cultures were maintained in a humidified incubator at 37°C and 5% CO_2_. For *in vitro* experiments, BMS-690514 was dissolved in dimethyl sulphoxide at 50 *μ*mol l^−1^ as a stock solution and stored at −20°C. For *in vivo* studies, BMS-690514 was prepared fresh before each administration. It was dissolved in 1,2-propanediol and Tween 80 to a final 1,2-propanediol concentration of 40% and Tween 80 concentration of 10%.

### Clonogenic survival assays

First, a clonogenic assay was performed without irradiation and with various doses of BMS-690514 to demonstrate dose response and calculate the concentration of BMS-690514 to inhibit 50% of cells (IC50). To investigate the effect of BMS-690514 on the response of cell lines to radiation, a standard clonogenic assay was performed. Survival after radiation exposure was defined as the ability of cells to maintain clonogenic capacity and form colonies. Briefly, after incubation intervals of 12 h with BMS-690514 at a dose ranging from 20 to 500 nmol l^−1^, cells were washed and exposed to radiation at a dose ranging from 2 to 6 Gy using 200-kV X-rays, and then cells were trypsinised, counted, and seeded for colony formation in 35-mm dishes at 50–1000 cells per dish. After incubation intervals of 14 days, colonies were stained with crystal violet and manually counted. All colonies of 50 or more cells were then counted. The survival fraction (SF) was estimated according to the following formula: SF=number of colonies formed/number of cells seeded × plating efficiency of the control group. All experiments were performed in triplicate.

### Assay for tumour growth in athymic nude mice

*In vivo* experiments were carried out at the Institut Gustave Roussy under Animal Care license no 94-076-11 (Ministère de l’Agriculture). Female athymic nude mice (6–8 weeks of age) purchased from Janvier CERT (Le Genest St. Isle, France) were used. A549, H1975, and H1299 cells were collected in exponential growth phase and ∼3 × 10^6^ cells were injected s.c. into the flank area of 6- to 8-week-old female athymic nude mice on day 0. When tumours reached the appropriate size, mice were randomised into groups of six mice each and treated.

Three separate animal experiments were performed:

(a) BMS-690514 alone: Mice bearing tumours with a volume of 75–150 mm^3^ were randomly assigned to receive BMS-690514 (30 mg kg^−1^ day^−1^) or vehicle (1, 2-propanediol and Tween 80 in saline water). Treatment was administered once daily by oral gavage for 5 days.

(b) BMS-690514+IR: For BMS-690514 and radiation studies, mice bearing established tumours of a volume of 75–150 mm^3^ were randomised into groups of six to orally receive either vehicle or BMS-690514 (30 mg kg^−1^ per os quaque die (every day) × 5, × 1 week) once daily for the duration of the experiment. BMS-690514 or vehicle was administered with or without irradiation (6 Gy in a single administration on day 3). Localised irradiation was administered at a dose rate of 0.85 Gy min^−1^ using mouse jigs.

(c) IR+adjuvant BMS-690514: For H1299 and H1975 xenografts, BMS-690514 was given the first day after IR at a dose of 6 Gy, and from day 2 to day 12 according to a sequential schedule.

Mice were weighed, and tumour size was measured twice a week with an electronic caliper. Individual mice were followed up over 30 days after the beginning of the treatment. Tumour volume was estimated from two-dimensional tumour measurements using the following formula: 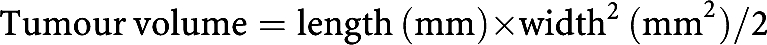


In each group (six mice per group), the relative tumour volume was expressed as the *V*_t_/*V*_o_ ratio (*V*_t_ as the mean tumour volume on a given day during the treatment and *V*_o_ as the mean tumour volume at the beginning of the treatment).

The experimental end point was taken as the time for the tumour to reach a relative tumour volume of five times that at the initiation of therapy (RTV5). When tumours reached RTV5, mice were killed. Overall survival was calculated using the Kaplan–Meier method. Growth delay was calculated by subtracting the average time for control tumours to grow five-fold in volume from the time required for treated tumours to increase in volume by the same amount from the first day of treatment.

### Immunohistochemistry

Histological assessment was carried out to evaluate the effects of BMS-690514 on tumour vasculature. Three animals were killed after the 3-day treatment with BMS-690514. Tumours were excised and, after fixing them in Finefix (Milestone, Italy), paraffin sections (4-*μ*m thick) were prepared. After xylene treatment and rehydration, immunohistochemistry of anti-CD34-positive murine endothelial cells (HyCult Biotechnology b.v., Uden, The Netherlands) was performed. Heat-induced epitope retrieval was achieved with Tris–EDTA (pH 8) at 98°C for 40 min. Endogenous peroxidase activity was quenched by treatment with 3% H_2_O_2_ for 10 min. The sections were placed in coverplates (Shandon, Pittsburgh, Pennsylvania, USA) and incubated with blocking serum Biogenex wash buffer (1:10 dilution; San Ramon, CA, USA) for 10 min. This step was followed by incubation with anti-mouse CD34 (1:20 dilution) diluted in blocking serum (1:10 dilution) for 1 h. Slides were then incubated with a rabbit anti-rat (1:400) (Southern Biotechnology, Birmingham, AL, USA) diluted in blocking serum (1:10 dilution). The following step was performed using a Rabbit PowerVision kit (ImmunoVision Technologies, Burlingame, CA, USA) for 20 min and diaminobenzidine for 10 min. Slides were counterstained with Mayer's hematoxylin and mounted with Pertex. Tumour necrosis was assessed by light microscopy. The number of microvessels per field was scored by averaging five field counts of three individual tumours for each group.

### X-ray irradiation

A 200-kV X-ray irradiator was used at a dose rate of 0.85 Gy min^−1^. For *in vivo* irradiation, radiation was given using mouse jigs designed to expose only the tumour bed to radiation.

### Statistical analysis

All descriptive statistics, including mean±s.d., were performed. A Student's *t*-test was used to evaluate differences between the control group and each treatment group in all *in vitro* studies. Kaplan–Meier curves were used for *in vivo* experiments and the log-rank test was used for statistical comparisons.

## Results

**Figure 1:**
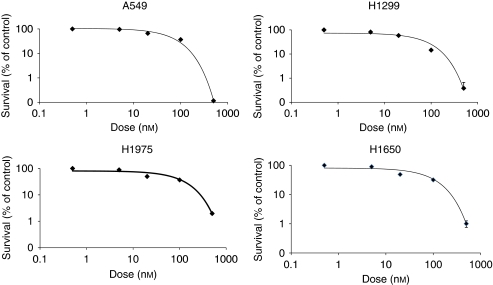
Anti-proliferative effect of BMS-690514 on NSCLC cell lines. A549, H1299, H1975, and H1650 cells were treated with the indicated concentrations of BMS-690514 for 12 h and then clonogenic survival assay was performed. Colonies were counted 14 days later. Clonogenic survival of cultures treated with BMS-690514 is normalised to that of untreated cells. One representative experiment is shown here.

The VEGFR and EGFR inhibitor, BMS-690514, decreases the survival of NSCLC cells lines, regardless of the EGFR mutational status at submicromolar concentrations. The effect of BMS-690514 on the proliferation of four NSCLC cell lines was determined by clonogenic assay after a 12-h incubation with BMS-690514 at doses ranging from 5 to 500 nmol l^−1^. Clonogenic surviving fraction was significantly reduced in all cell lines including the EGFR mutation (T790M)-harbouring cell line (H1975), a mutation that confers resistance against erlotinib (Yun *et al*, 2008). This effect was obtained at an IC50 value of ∼20 nmol l^−1^ for A549 and H1975 cell lines; at ∼10 nmol l^−1^ for the H1650 cell line; and at ∼40 nmol l^−1^ for the H1299 cell line (Figure 1), and was dose dependent. These data are consistent with those reported by a previous study that showed that these concentrations led to a significant decrease in EGFR phosphorylation (De la Motte Rouge *et al*, 2007).

### IR cell killing is not affected by BMS-690514

**Figure 2:**
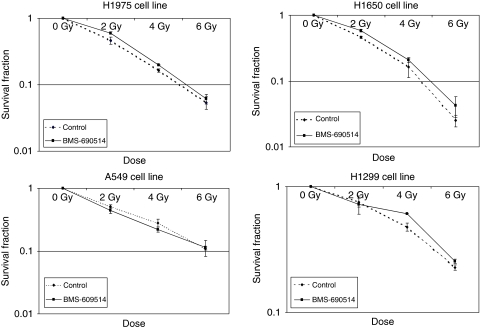
Anti-proliferative effect of BMS-690514 combined with radiation on NSCLC cell lines. H1975, H1650, H1299, and A549 cells were incubated with (-▪-) or without (···⧫···) BMS-690514 (20 nmol l^−1^) for 12 h and then treated with radiation at a gradual dose; clonogenic assay was then performed. Colonies were counted 14 days later. Clonogenic survival of cultures treated with radiation is normalised to that of unradiated cells. Columns represent mean of triplicate wells and bars represent s.d. values.

No potential *in vitro* enhancement of radiation cell killing was observed with BMS-690514 in NSCLC cells at a dose of 20 (Figure 2) or 500 nmol l^−1^ (data not shown). Clonogenic survival was not different in the four NSCLC cell lines treated with BMS-690514, radiation, or both.

### BMS-690514 alone inhibits growth of NSCLC xenograft regardless of EGFR mutational status

**Figure 3:**
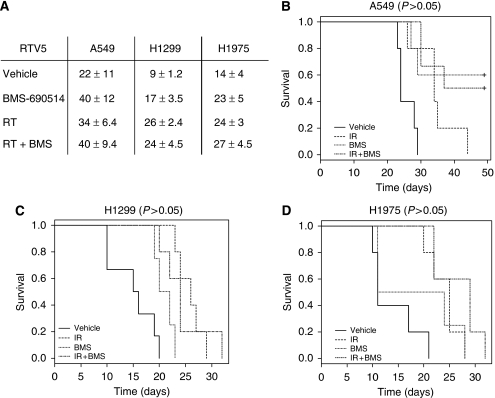
BMS-690514 does not enhance the efficacy of radiotherapy in NSCLC xenografts in concomitant sequence. (**A**) Effects of BMS-690514, radiation (RT), or both on RTV5 (days). RTV5 was calculated as the time for the tumour to reach a relative tumour volume that is five times of that at the initiation of therapy. Kaplan–Meier survival curves of mice inoculated with A549 cells (**B**), H1299 cells (**C**), and HT1975 cells (**D**) treated with vehicle (—) or BMS-690514 (**····**); 30 mg kg^−1^; p.o.; (from day 1 to day 5) alone or IR alone (- -); 6 Gy on day 3 or both (- - ), (concurrent sequence). *n=*6 per group. Statistical analysis: (**B**) A549: combination *vs* IR, *P*=0.12; combination *vs* BMS-690514, *P*=0.96; (**C**) H1299: combination *vs* IR, *P*=0.82; combination *vs* BMS-690514, *P*=0.08; (**D**) H1975: combination *vs* IR, *P*=0.10; combination *vs* BMS-690514, *P*=0.08.

Continuous oral dosing of A549, H1299, and H1975 tumour-bearing mice with BMS-690514 for 5 days resulted in tumour growth inhibition. Figure 3A shows that BMS-690514 increases survival in A549 tumour-bearing mice (*P*=0.02), H1299 tumour-bearing mice (*P*=0.02), and H1975 tumour-bearing mice (*P*=0.1), compared with mice treated with vehicle. The time taken to reaching a relative A549 tumour volume equivalent to five times that at the start of the treatment (RTV5) was 40±12 days with BMS-690514 compared with 22±11 days with vehicle (*P*=0.02). Similar data were observed for H1299 tumour-bearing mice: RTV5 was 17±3.5 days with BMS-690514 compared with 9±1.2 days with vehicle (*P*=0.02). RTV5 was 23±5 days in H1975 tumour-bearing mice administered BMS-690514 compared with 14±4 days for mice treated with vehicle (*P*=0.15).

### BMS-690514 has sequence-dependent synergistic anti-tumour activity with radiation

To evaluate the potent interaction between BMS-690514 and radiation, tumour-bearing mice were treated with either BMS-690514 or radiation, or both, in concomitant schedule.

The potential of BMS-690514 to enhance the efficacy of radiotherapy in NSCLC xenografts was first investigated in a concurrent schedule, in which 30 mg kg^−1^ BMS-690514 was given orally once a day from day 1 to day 5 and 6-Gy radiation was administered at day 3. No increase in survival was observed in mice treated with both treatments (Figure 3B–D).

**Figure 4:**
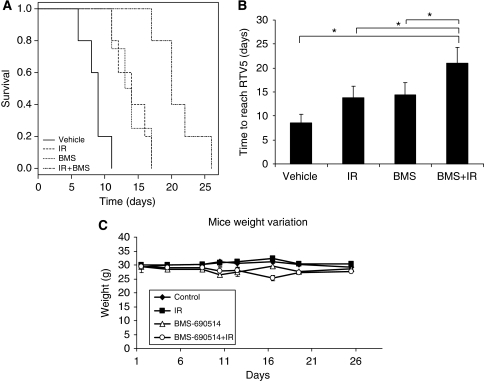
BMS-690514 enhances the efficacy of radiotherapy in NSCLC xenografts in sequential manner. (**A**) Kaplan–Meier survival curves of H1299 tumour-bearing mice treated with vehicle (**·-·-·-·**) or BMS-690514 (**·-·-·-·**); (30 mg kg^−1^ p.o. from day 2 to day 12) alone or with IR alone (- -); (6 Gy on day 1), or with both (- - ); adjuvant sequence. *n=*6 per group. Statistical analysis: combination *vs* vehicle, *P*=0.002; combination *vs* IR; *P*=004; combination *vs* BMS-690514, *P*=0.007; IR *vs* vehicle, *P*=0.005; BMS-690514 *vs* vehicle, *P*=0.01. (**B**) Influence of BMS-690514 (30 mg kg^−1^, p.o. from day 1 to day 12) and/or IR treatment on time to achieve RTV5. Data are shown as mean values (±s.d.) with *n=*6 per group. ^*^, *P*<0.05; (Student's *t* test); RTV5 was calculated as the time for the tumour to reach a relative tumour volume five times of that at the initiation of therapy. Growth delay was calculated by subtracting the average time for control tumours to grow five-fold in volume from the time required for treated tumours to increase in volume by the same amount from the first day of radiation. (**C**) Mice weight in untreated and treated mice.

**Figure 5:**
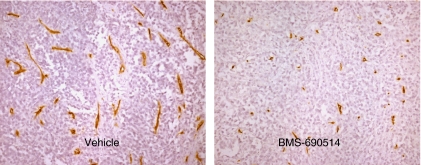
BMS-690514 induces a trend towards reduced tumour perfusion. CD-34 staining of perfused vessels in a tumour treated with one daily fraction of vehicle or BMS-690514 (30 mg kg^−1^ day^−1^ for 3 days).

While on a concurrent schedule, no enhancement of radiation was observed; BMS-690514 markedly enhances the anti-tumour effect of radiation in a sequential manner (Figure 4). H1299 xenograft-bearing mice, treated with a 30-mg kg^−1^ oral dose of BMS-690514 from day 2 to day 12 after the completion of radiation (6 Gy at day 1), exhibited a significant increase in survival (median survival=20 days), compared with mice treated with radiation alone (median survival=13.5 days, *P*=0.004) or BMS-690514 alone (median survival=14 days, *P*=0.07). Furthermore, the association leads to an increase in time to reach RTV5 as compared with mice treated with BM-690514 alone or radiation alone (Figure 5B). The association was well tolerated (Figure 5C). Sequential scheduling radiation followed by 30-mg kg^−1^ BMS-690514 treatment for 12 days induced a mean body weight loss of 7%±2. This was not different from that induced by 12 days of treatment with 30-mg kg^−1^ BMS-690514 alone. These results were also observed for H1975 xenograft-bearing mice: mice treated with a 30-mg kg^−1^ oral dose of BMS-690514 from day 2 to day 12 after completion of radiation (6 Gy at day 1) exhibited a significant increase in survival (median survival=20 days) compared with mice treated with radiation alone (median survival=14 days, *P*=0.005) or BMS-690514 alone (median survival=9 days, *P*=0.05).

### BMS-690514 decreases vascularisation in xenografts

As shown in Figure 5, BMS-690514 could decrease vascularisation in H1299 xenografts as compared with untreated H1299 xenografts, suggesting an anti-angiogenic effect of BMS-690514. After 3 days of BMS-690514 administration (30 mg kg^−1^ day^−1^), the proportion of tumour vessels seemed to be lesser than that in vehicle-treated controls and a qualitative reduction in CD34-positive vessel area was observed.

## Discussion

Patients with locally advanced NSCLC have a poor outcome with a 5-year survival rate of 15%. As chemoradiotherapy for these patients has a modest impact on survival, associations of new targeted therapies with radiation are needed. As EGFR and VEGFR expressions were shown to be important for NSCLC cancer, there is significant rationale behind the treatment of these tumours with BMS-690514. In this study, we showed that the initial inhibition of EGFR and VEGFR by a small inhibitor of tyrosine kinase used as a stand-alone treatment could induce an anti-tumoural effect on several NSCLC xenografts. Chronic administration of BMS-690514 (30 mg kg^−1^ day^−1^) is well tolerated in athymic mice (Figure 4C) and produces significant inhibition of tumour growth (Figure 3). These results are consistent with previous *in vitro* data that reported a cytotoxic effect on NSCLC cell lines (De la Motte Rouge *et al*, 2007). Interestingly, these effects were observed even on T790M-harbouring tumours that confer resistance to an EGFR inhibitor such as Erlotinib (Figure 3). We then showed that this double tyrosine kinase blockade could enhance the anti-tumoural effect of radiotherapy in a sequential manner (Figure 4).

We observed that BMS-690514 had no effect on radiation response in *in vitro* assays but led to a marked tumour growth delay when used sequentially with IR *in vivo*. Although no radiosensitisation effect was observed in the concomitant schedule, a strong synergistic activity was shown in adjuvant sequence (Figure 4). The positive interaction observed when BMS-690514 is used as an adjuvant to radiotherapy supports an important role for VEGF-mediated pathway in the tumour response to radiotherapy *in vivo*. The anti-tumour activity of radiation could be influenced by many factors, such as intrinsic tumour radiosensitivity, altered capacity for DNA repair, and the VEGF pathway. Some findings have shown that the induction of VEGF by irradiation contributes to the protection of tumour blood vessels from radiation-mediated cytotoxicity, and thereby to tumour radioresistance (Gorski *et al*, 1999). Furthermore, radiation therapy resulted in an increased expression of both VEGFR-2 and EGFR in lung tumours, leading to tumour proliferation and survival (Shibuya *et al*, 2007).

Adjuvant inhibition of angiogenesis, that is, application of anti-angiogenic compounds after radiotherapy has been completed, is suggested to prolong the dormancy of residual tumour cells by inhibiting the formation of the new blood vessels that are required for regrowth of tumour cells that survived irradiation (Kerbel, 2008). Vascular endothelial growth factor seems to be expressed as the principal pro-angiogenic factor for early-stage cancers (Ferrara, 2004), and tumour progression, including progression after curative treatment, is often associated with an altered expression of other pro-angiogenic factors. This concept is supported by some studies that show that tumour blood flow increases after irradiation, probably contributing to the regrowth of cancer because certain cells survived irradiation (Hori *et al*, 2008).

The questions of optimal scheduling have been addressed in limited studies combining radiotherapy with a number of anti-angiogenic agents (Williams *et al*, 2004; Dings *et al*, 2007; Shibuya *et al*, 2007) A study using concomitant PTK787/ZK222584 failed to show enhancement over radiotherapy alone, and study results showed that drug therapy after irradiation was optimal (Zips *et al*, 2005). A study with Vandetanib reported that when radiation therapy is combined concomitantly with VEGFR2 and EGFR blockade, a significant enhancement of anti-angiogenic, anti-vascular, and anti-tumour effects is seen in an orthotopic model of lung cancer (Kerbel, 2008). Another previous study with Vandetanib in a Calu-6 tumour model showed that sequential administration with Vandetanib after radiotherapy was optimal, although concurrent treatment with Vandetanib and radiotherapy was better than treatment with either alone (Williams *et al*, 2004). Other tyrosine kinase inhibitors with anti-angiogenic effects, such as SU5416 (a VEGFR inhibitor) and SU6668 (an inhibitor of VEGF, fibroblast growth factor, and platelet-derived growth factor receptors), also enhance the anti-tumour effects of fractionated irradiation independent of drug sequencing (Ning *et al*, 2002).

The radiopotentiating effect of BMS-690514 was reduced when BMS-690514 was administered concomitantly with radiation as compared with a sequential schedule. After 3 days of BMS-690514 administration (30 mg kg^−1^ day^−1^), the proportion of abnormal tumour vessels seemed to be lesser than that in vehicle-treated controls and a qualitative reduction in CD34-positive vessel area was observed (Figure 5). Hence, this reduced perfusion could induce an increase in hypoxic fraction within treated tumours that could potentially affect the tumour response to radiotherapy when an anti-angiogenic compound is administered before or during radiotherapy. The reduced perfusion observed as a consequence of BMS-690514 administration may inhibit this effect, thereby limiting the potential for reoxygenation and the efficacy of the fractionated radiotherapy used. These observations are consistent with previous reports of reduced tumour vascular permeability/perfusion after Vandetanib treatment, a VEGFR and EGFR inhibitor (Checkley *et al*, 2003).The results from the study with Vandetanib showing that the schedule of concomitant association of Vandetanib results in poor enhancement of radiation were attributed to decreased perfusion in the concurrent schedule such that radiotherapy was impaired (Williams *et al*, 2004). However, the effect of the sequence could be related to EGFR blockade, as these results were not observed with SU5416 (a VEGFR inhibitor) and SU6668 (an inhibitor of VEGF, fibroblast growth factor, and platelet-derived growth factor receptors).

The type of cell lines tested may also influence the sequence dependency of the findings with concurrent irradiation, as comparisons between models strongly and weakly expressing EGFR show that EGFR ‘strong’ cell lines exhibit both increased tumour growth delay after exposure to IR and a dual EGFR–VEGFR2 inhibition with paradoxical superiority of concomitant versus adjuvant sequencing in EGFR ‘weak’ cell lines (Gustafson *et al*, 2008).

A preclinical evaluation of the combination of ionising radiation and 4 h after the last radiation dose showed that a vascular disrupting agent, ZD6126, combined with the anti-EGFR Gefitinib did enhance *in vivo* tumour growth delay in NSCLC models (Raben *et al*, 2004).

Altogether, the sequence dependency effects of the combination of anti-vascular agents and anti-EGFR inhibitors on ionising radiation may differ depending on models and drugs. However, there is consistency regarding the beneficial effect of adjuvant treatment. Our data are in line with these findings. Given the fact that this type of scheduling offers minimal toxicity hazard compared with concurrent treatment, adjuvant treatment should be the optimal treatment in a clinical setting.

In conclusion, these data show that inhibition of both the VEGF and EGFR pathways demonstrates efficacy in NSCLC xenografts and that combining such agents with radiation could improve the anti-tumour activity of radiation alone. However, scheduling seems to be an important factor to be taken into account to establish optimal effect. This study supports the development of BMS-690514 in NSCLC as an adjuvant to radiotherapy in a clinical.
